# A survey on cultivable heterotrophic bacteria inhabiting a thermally unstratified water column in an Atlantic Rainforest lake

**DOI:** 10.7717/peerj.478

**Published:** 2014-08-26

**Authors:** Cláudia I. Lima-Bittencourt, Patrícia S. Costa, Mariana P. Reis, Alexandre B. Santos, Francisco A.R. Barbosa, Jean L. Valentin, Fabiano L. Thompson, Edmar Chartone-Souza, Andréa M.A. Nascimento

**Affiliations:** 1 Departamento de Biologia Geral, Instituto de Ciências Biológicas, Universidade Federal de Minas Gerais, Belo Horizonte, Minas Gerais, Brazil; 2 Laboratório de Microbiologia, Instituto de Biologia, Universidade Federal do Rio de Janeiro, Rio de Janeiro, Brazil

**Keywords:** Tropical freshwater lake, Bacteria; 16S rRNA

## Abstract

Due to the importance of heterotrophic bacteria in biogeochemical cycles and their influence on water quality, many studies have assessed the composition of the bacterial community. Most of these were made in temperate freshwaters. Eighteen heterotrophic bacteria communities distributed over time and space in the water column of Carioca Lake, not exposed to anthropogenic activities, were analyzed to characterize their composition. A polyphasic approach was used, including 16S rDNA restriction analysis, 16S rRNA gene sequence analysis, BIOLOG Ecoplates and statistical methods. The physiological profiles among the 18 microbial communities were diverse. Clustering analysis and the metabolic fingerprint of the Biolog Ecoplate^TM^ system data separated the communities based on temporal scale. A set of 673 isolates were recovered on high nutrient medium. The 673 isolates obtained yielded 360 Amplified Ribosomal DNA Restriction Analysis (ARDRA) Operational Taxonomic Units (OTUs). Most (313) of the ARDRA patterns, OTUs, were from isolates obtained in a single sampling point, in temporal and spatial scales, indicating changes in the bacterial community. A subset of representative isolates for each ARDRA OTU was identified by 16S rRNA gene fragment sequencing and categorized into five phyla, *Proteobacteria*, *Actinobacteria*, *Bacteroidetes*, *Firmicutes*, and *Deinococcus*-*Thermus*, represented by 38 genera. The results of this work contribute to a better understanding about the phylogeny of tropical freshwater heterotrophic bacteria.

## Introduction

Heterotrophic bacteria are widely recognized as essential performers in biogeochemical cycling, from fueling the food web to elemental fluxes (Cooney & Simon, 2009). It is known that environmental conditions such as water nutrient concentration, pH, and temperature are able to change the spatial and/or temporal dynamics of prokaryotes playing a significant role in shaping freshwater bacterial communities (Rubin & Leff, 2007). Changes in composition of the bacterial community are excellent biological indicators because they can reflect the water quality and the diversity of the ecosystem (Sartory, Gu & Chen, 2008). Indeed, a parameter commonly used to assess the general microbiological quality of drinking water is their abundance, which depends on the trophic status of the water body, ranging from 0.5 to 1 × 10^6^ cells/ml in oligotrophic environments to 10 × 10^6^ cell/ml in eutrophic environments (Cotner & Biddanda, 2002). Moreover, heterotrophic bacteria impact on cyanobacterial growth, which can cause harmful effects on human and animal health (Bertilsson, Granéli & Carlsson, 2004; Berg et al., 2009). However, the identity of bacteria that inhabit freshwater of relatively pristine environments has not been extensively explored so far.

Over the last decades, many freshwater studies have used molecular approaches to examine the bacterial community composition of lentic environments, particularly in temperate regions. Yet, little is known about the bacterial composition of tropical lakes despite the fact that they constitute a significant proportion of world freshwater resources (Sorrento, 2012).

A meta-analysis of published upper-layer freshwater lake 16S rRNA gene sequences revealed that most bacteria fall into five phyla: *Proteobacteria* especially *Betaproteobacteria*, *Actinobacteria*, *Bacteroidetes*, *Cyanobacteria*, and *Verrucomicrobia*. The research, published by Newton et al. (2011), revealed that these phyla make up about 97% of the >11,400 sequences analyzed, a finding that is in agreement with Zwart et al. (2002) and Humbert et al. (2009). In addition, 16S rRNA gene sequences from *Gamma-proteobacteria* are poorly recovered from freshwater (Zwart et al., 2002).

Traditionally the culture-based approach has been employed for understanding the physiological potential, but does not provide insight into the diversity of microbial communities in natural environments: over 99% of microorganisms cannot be cultured in the laboratory (Pace et al., 1985). Hence, most research on freshwater bacterial communities has focused on diversity using a culture-independent approach, a method with limited ability to detect heterotrophic freshwater bacteria (Berg et al., 2009). Indeed, previous comparative studies using culture-dependent and independent approaches revealed that the diversity and phylogeny of bacteria of a freshwater sample were different and that the latter method failed to detect most bacterial isolates (Pearce et al., 2003; Vaz-Moreira et al., 2011).

Currently, little is known about the ecology, physiology and community composition of cultivable heterotrophic bacteria from tropical lakes. Therefore, to gain insight into this knowledge gap we used a cultivation-dependent approach and techniques based on 16S rRNA gene sequencing, followed by a multiple eco-physiological characterization strategy.

## Methods

### Study area

In 1944, the Parque Estadual do Rio Doce (PERD; Fig. S1) was established as an Atlantic Forest conservation area in Brazil. PERD makes up the largest remaining area of the Atlantic Forest biome (36,000 ha of forests) in the state of Minas Gerais and for this reason has been subjected to long-term ecological research. Its lake system, comprising 51 lakes, occupies 9.8% of the total area (Bezerra-Neto, Briguenti & Pinto-Coelho, 2010). The object of the present study is the Carioca Lake, a shallow (11.8 m maximum depth) and small lake (14.1 ha) (Brito & Maia-Barbosa, 2009). Its water circulates from May/June to August (Henry & Barbosa, 1989), remaining stratified for the rest of the year. It is a warm monomictic lake hydrologically isolated from the original main drainage, the Doce River, which is approximately 40 m deeper than the lakes formed in its paleo-canal (De Meis & Tundisi, 1997).

### Water sampling

Triplicate water samples (500 mL) were collected from three points 100 m distant from each other across a euphotic gradient from a limnetic zone (Carioca Lake) with the help of van Dorn bottles. Collections took place in the dry season, more precisely in the months of June and August 2007. Samples were taken from the water column at different degrees of light penetration (100%, 10%, and 1%) as determined by Secchi disk. Due to the size of the lake we considered it relevant to do several samplings, covering distinct points and horizontal and vertical gradients of the limnetic zone in June and August. In this way, we aimed to improve the representativeness of the lake’s cultivable heterotrophic bacterial community.

Selected physical and chemical variables were measured at three points in the euphotic gradient to assess the water conditions. Water temperature, pH, turbidity and dissolved oxygen concentration (DO) were measured *in situ* with a multiprobe (Horiba, model U-22). Concentrations of total nitrogen (TN), ammonium $({\mathrm{NH}}_{4}^{+}{\unicode{x2013}}\mathrm{N})$, nitrite $({\mathrm{NO}}_{2}^{2-}{\unicode{x2013}}\mathrm{N})$, nitrate $({\mathrm{NO}}_{3}^{2-}{\unicode{x2013}}\mathrm{N})$, total phosphorus (TP), soluble reactive phosphorus $({\mathrm{PO}}_{4}^{3-}{\unicode{x2013}}\mathrm{P})$ were measured according to Mackereth, Heron & Talling (1978) and Golterman, Clymo & Ohnstad (1978), respectively. For chlorophyll *a* concentration determination, pigment was extracted with 90% acetone (Lorenzen, 1967). The readings were performed with a Shimadzu model UV 1700 spectrophotometer.

All necessary permits were obtained from the Instituto Estadual de Florestas (no. 018/2008) for the field studied.

### Microbial metabolic diversity

The BIOLOG (BIOLOG Inc., USA) plates were originally designed to identify bacterial isolates. However, they have been found useful in bacterial community studies and have been widely used to characterize these communities from various environments, including those that are freshwater (Garland & Mills, 1991). The BIOLOG Ecoplate system allows the fast growing of heterotrophic bacteria, providing a simpler, more rapid and reproducible approach to assess the shifts in metabolic profiles of bacterial communities. Thus, to obtain more insight into the diverse carbon sources used by heterothrophic bacteria the pattern of carbon (C) source utilization was directly obtained from water samples, which could reflect the metabolic diversity of the bacterial isolates retrieved from Carioca Lake. Community-level physiological profiles (CLPP) were measured using a BIOLOG 96-well Ecoplate following the manufacturer’s protocol. This system is composed of 3 × 31 single carbon sources and a water blank well. One hundred twenty µl of sample water was inoculated into each well of the Ecoplate and incubated at 28 °C in the dark. Color development was measured at OD_590_ every 24 h for five days using an ELISA plate reader (BIO-RAD Model 3550 Microplate Reader). The absorbance in the water blank was subtracted from the absorbance readings of all other wells. Negative optical density values were set to zero. Plate readings at 72 h of incubation were used for the assessment of bacterial functional diversity and statistical analyses because this represented the optimal range of optical density readings (0.3–1.0 abs).

### Ecoplate data analysis

Raw OD_590_ values were corrected for the microbial activity for each microplate, expressed as the average well-color development (AWCD) and calculated as follows:

\begin{eqnarray*} \mathrm{AWCD}=\sum {\mathrm{OD}}_{i}/31 \end{eqnarray*}

where OD_*i*_ is the optical density value from each well. Richness (the number of oxidized C substrates) and the Shannon–Weaver index (the richness and evenness of response) were calculated using an OD of 0.25 as the threshold for positive response (Garland, 1996). Shannon’s diversity index was calculated as: *H*′ = ∑*p_i_*(ln *p_i_*) where *p_i_* is the ratio of the activity on each substrate (OD_*i*_) to the sum of activities on all substrates (∑OD_*i*_). Shannon’s evenness was calculated as: *E* = *H*′/ln *R* where *H*′ is Shannon’s diversity and *R* is substrate richness.

According to Legendre & Legendre (2000), ordered data do not have to be transformed before they are analyzed numerically. Thus, the Ecoplate data were normalized and transformed logarithmically before multivariate analyses were applied. The 72 h absorbance values were normalized by the AWCD, as recommended by Garland (1996): ${\mathrm{OD}}_{k}=\frac{{\mathrm{OD}}_{k}}{\frac{\sum {\mathrm{OD}}_{i}}{31}}$ where OD_*k*_ represents the normalization data of well *k*, OD_*k*_ is the absorbance reading of well *k*, and the denominator in this equation represents the AWCD. This transformation is likely to normalize skewed data (Legendre & Legendre, 2000). A natural logarithmic transformation was used in this study: OD′ = ln(OD_*k*_ + 1) where OD′ represents the value of the transformed data.

## Statistical Analysis

Correspondence analysis (CA) was performed as described by Benzécri (1973). It is a geometric technique for displaying the rows and columns of a two-way contingency table as points in a low-dimensional space, such that the positions of the rows (here 30 Ecoplate substrates) and columns (here 18 sampled waters) are consistent with their associations in the table. The goal is to have a global view of the data that is useful for interpretation. CA can also be applied to binary data, which is of great interest in the present case where response of communities to each substrate can be represented by 1 and 0. The substrates 2-hydroxy benzoic acid and xylose were excluded from the analysis as they did not add information and increased the variance.

### Bacterial isolation

Bacteria were isolated by plating 100 µl of the water samples collected directly on PTYG agar plates (0.5% peptone, 0.5% tryptone, 0.5% yeast extract, 1.0% glucose, 0.06% MgSO_4_, 0.006% CaCl_2_, 1.5% agar) and incubated at 28 °C for up to seven days. The resulting colonies, with different morphologies (size, shape, surface, color, texture, and elevation), were re-suspended in saline (0.85%, w/v), vortexed and repeatedly streaked on the same medium to accomplish their purification, prior to molecular analyses. The isolates were stored in glycerol at −70 °C until further use. Isolates were named according to each point of the limnetic zone (A, B, and C), specific euphotic gradient (1%, 10%, and 100% of light penetration) and month of collection (J for June and A for August) from which they were retrieved, in this order (e.g., LimA-1-J, LimB-10-A, and LimC-100-J).

### DNA extraction and 16S rRNA gene amplification

Genomic DNA of the isolates was purified as described previously (Sambrook & Russell, 2001). The 16S rRNA gene was amplified by touchdown PCR according to Pontes et al. (2009), using the conserved primer set PA (5′-TCCTGGCTCAGATTGAACGC-3′) (modified from Kuske, Barns & Busch, 1997) and U2 (5′-ATCGGYTACCTTGTTACGACTTC-3′) (Lu et al., 2000).

### Amplified ribosomal DNA restriction analysis (ARDRA)

ARDRA was performed to analyze bacterial diversity and to compare the composition of the communities from each point sampled. 16S rRNA gene was amplified with the PA and U2 primers and then digested separately with two restriction enzymes (AflIII and AluI; New England Biolabs) that recognize sequences of six nucleotides, according to the supplier’s recommendations. Restriction fragments were separated on 2.5% agarose gels in 40 mM Tris, 20 mM acetic acid, and 1 mM EDTA (TAE) buffer, pH 8. After electrophoresis at 75 V for 2.5 h, the gels were observed and photographed. Restriction fingerprints were analyzed using BioNumerics version 6.0 software (Applied Maths, St. Martens-Latem, Belgium). Digitized gel images were converted and normalized using the 1 Kb Plus DNA Ladder (Invitrogen). A band-matching algorithm (band-matching tolerance of 1.0%) was used to calculate pairwise similarity matrices with the Dice coefficient. Cluster analyses of similarity matrices were performed by unweighted pair group method using arithmetic averages (UPGMA). For each ARDRA pattern, the 16S rRNA gene of one to three isolates was sequenced, thus minimizing the number of sequence reactions that had to be performed.

### Sequencing and phylogenetic analysis

The partial 16S rRNA gene sequence was obtained using the primers 8F (5′-GAGTTTGATYMTGGCTCAG-3′) and 907R (5′-CCGTCAATTCMTTTRAGTTT-3′) (Lane, 1991). Sequencing reactions were performed with the BigDye Terminator v3.1 Cycle Sequencing kit (Applied Biosystems) and an ABI 3130 sequencer (Applied Biosystems) according to the manufacturer’s instructions. The 16S rRNA gene sequences were assembled using Linux programs Phred/Phrap/Consed (http://www.phrap.org/phredphrapconsed.html), and compared with available database using the BLASTn search tool from GenBank (http://www.ncbi.nlm.nih.gov/) to identify the closest relatives of the sequences. Phylogenetic relationships were inferred with the neighbor-joining algorithm (Saitou & Nei, 1987) using the ARB (version 5.3) software package and SSU Ref 115 SILVA sequence database (Ludwig et al., 2004; Pruesse et al., 2007). The nucleotide sequences generated were deposited in the GenBank database with accession numbers JF900768–JF901324.

### Bacterial community analysis

The Unifrac metric method (http://bmf.colorado.edu/unifrac) was used to compare bacterial communities from each point of the limnetic zone with those from every euphotic gradient by using phylogenetic information (Lozupone, Hamady & Knight, 2006). The phylogenetic data were used to compare bacterial communities, testing statistical differences among all samples, with UPGMA. The Cluster Environments function of Unifrac was used to determine which environments in the tree had similar bacterial communities. Jackknife analysis was used to test the reliability of the Unifrac dataset.

The rarefaction curve was estimated for communities’ sequence data. Bacterial isolate sequence coverage was calculated using the equation *C* = [1−(*n_i_*/*N*)] × 100, where *n_i_* represents the number of genera represented by a bacterial isolate sequence and *N* represents the total number of sequences in the community.

## Results

### Environmental characterization

The physicochemical and biological parameters of the water column are presented in Table 1. There was little variability in temperature along the euphotic gradient during the sampling periods, which indicated a thermally unstratified water column. The June collection showed slightly acidic water, with pH values decreasing with depth, whereas in August the pH remained close to neutrality and varied little with depth. While turbidity was ≤7 nephelometric turbidity units (NTU) in August, no vertical variation was detected in June. Despite being a thermally unstratified lake, bottom waters of Carioca Lake revealed a slight decrease in oxygen concentration in both sampling periods when compared to top waters, and particularly in August. Total phosphorous (TP) and nitrogen (TN) concentrations increased with depth in both sampling periods. The trophic status of the water was mesotrophic during the sampling periods, a classification based on TP levels according to the model of Salas & Martino (1991). The highest phytoplankton production (biomass), as determined by chlorophyll *a* concentration, was observed at the layer corresponding to 1% of surface irradiance. This observation demonstrates light inhibition at the surface as pointed out for this lake by Barbosa & Tundisi (1980).

**Table 1:** Environmental parameters obtained in the water column from Carioca Lake in 2007.

Environmental parameters	Sample period					
	June			August		
Light penetration	100%	10%	1%	100%	10%	1%
Depth (m)	0	1	3	0	1.5	4.5
pH	7.3	6.3	5.6	7.4	7.7	7.3
Temperature (°C)	23	22.4	22	23.6	23	21.6
Turbidity (NTU)	7	7	7	2	2	7
DO (mg L^−1^)	9.1	8.7	7.3	8.4	8.3	6.8
TP (µg/L)	18.1	19.1	25.6	25.4	26.8	34.6
${\mathrm{PO}}_{4}^{3-}$–P (µg/l)	1.2	5.9	2.2	1.9	ND	3.9
TN (µg/l)	370.9	354.7	404.5	201.4	221.6	365.6
${\mathrm{NH}}_{4}^{+}{\unicode{x2013}}\mathrm{N}$ (µg/l)	121.9	114.6	112.7	43.1	41.6	22.8
${\mathrm{NO}}_{3}^{2-}{\unicode{x2013}}\mathrm{N}$ (µg/l)	32.2	38.5	32.2	30.1	47.6	43.5
${\mathrm{NO}}_{2}^{2-}{\unicode{x2013}}\mathrm{N}$ (µg/l)	1.7	2.1	1.6	1.6	2.3	1.4
Chlorophyll *a*	55.1	58.8	62	25.7	32.6	96.6

**Notes.**

DO dissolved oxygen concentration TP total phosphorus ${\mathbf{PO}}_{\mathbf{4}}^{\boldsymbol{3- }}$**–P** soluble reactive phosphorus TN total nitrogen ${\mathbf{NH}}_{\mathbf{4}}^{\boldsymbol{+}}$**–N** ammonium ${\mathbf{NO}}_{\mathbf{2}}^{\boldsymbol{2- }}$**–N** nitrite ${\mathbf{NO}}_{\mathbf{3}}^{\boldsymbol{2- }}$**–N** nitrate

### Physiological profiling of microbial community

The large number of investigated isolates in this study (673) makes it very laborious to test carbon source utilization for each isolate. Significant differences (*p* < 0.05) were found in R, AWCD, and *H*′ between the sampling periods. In contrast, R, AWCD, and *H*′ were not significantly different (*p* > 0.05) at each point of the horizontal and vertical gradients of the limnetic zone in June and August (Table 2).

**Table 2:** Carbon source utilization by bacterial communities from Carioca Lake and average well-color development (AWCD), richness (R), Shannon-Weaver index (H′) and evenness (E) calculated on carbon substrate used in Ecoplate.

Carbon sources	Sites sampled																	
	June									August								
	Lim A			Lim B			Lim C			Lim A			Lim B			Lim C		
	100%	10%	1%	100%	10%	1%	100%	10%	1%	100%	10%	1%	100%	10%	1%	100%	10%	1%
Pyruvic Acid Methyl Ester	+	+	+	+	+	+	+	+	+	+	+	+	+	+	+	+	+	+
Tween 40	+	+	+	+	+	+	+	+	+	+	+	+	+	+	+	+	+	+
Tween 80	+	+	+	+	+	+	+	+	+	+	+	+	+	+	+	+	+	+
Ciclodextrin	+	+	+	+	+	+	+	+	+	+	+	+	+	+	+	+	+	+
Glycogen	+	+	+	+	+	+	+	+	+	+	+	+	+	+	+	+	+	+
D-Cellobiose	+	+	+	+	−	+	+	+	+	+	+	+	−	+	+	+	+	+
*α*-D-Lactose	+	−	−	−	−	−	+	−	−	+	+	−	−	+	+	−	+	+
*β*-Methyl-D-Glucoside	+	+	+	+	+	+	+	+	+	+	+	+	+	+	+	+	+	+
D-Xylose	−	−	−	−	−	−	−	−	−	−	+	−	−	+	−	−	−	−
I-Erythritol	+	−	+	+	+	+	+	+	+	+	+	+	+	+	+	+	+	−
D-Mannitol	+	+	+	+	+	+	+	+	+	+	+	+	+	+	+	+	+	+
N-Acetyl-D-Glucosamine	+	+	+	+	+	+	+	+	+	+	+	+	+	+	+	+	+	+
D-Glucosaminic Acid	+	+	+	−	+	+	+	+	+	+	−	+	+	+	−	−	+	+
Glucose-1-Phosphate	+	+	+	+	−	+	+	+	+	+	+	+	+	+	+	+	+	+
D,L-*α*-Glycerol Phosphate	+	−	+	−	−	+	+	−	+	+	+	+	+	+	+	+	+	+
D-Galactonic Acid *γ*-Lactone	+	−	+	+	+	+	+	+	+	+	+	+	+	+	+	+	+	+
D-Galacturonic Acid	−	+	+	+	+	+	+	+	+	+	+	+	−	+	+	+	+	+
2-Hydroxy Benzoic Acid	−	−	−	−	−	−	−	−	−	−	−	−	−	−	−	−	−	−
4-Hydroxy Benzoic Acid	+	+	+	+	−	+	+	+	+	+	+	+	+	+	+	+	+	+
*γ*-Hydroxybutyric Acid	−	−	−	−	−	−	−	−	−	+	+	+	−	+	−	−	+	+
Itaconic Acid	−	+	+	+	+	−	+	+	+	+	+	+	+	+	+	+	+	+
*α*-Ketobutyric Acid	−	−	−	−	+	+	+	−	−	−	+	+	+	+	+	+	+	+
D-Malic Acid	+	+	+	+	+	+	+	+	+	+	+	+	+	+	+	+	−	+
L-Arginine	+	+	+	+	+	+	+	+	+	+	+	+	+	+	+	+	+	+
L-Asparagine	+	+	+	+	+	+	+	+	+	+	+	+	+	+	+	+	+	+
LPhenylalanine	+	+	+	+	−	+	+	+	+	+	+	+	+	+	+	+	+	+
L-Serine	+	+	+	+	+	+	+	+	+	+	+	+	+	+	+	+	+	+
L-Threonine	+	−	−	−	−	−	−	−	+	+	+	+	+	+	+	+	+	+
Glycyl-LGlutamic Acid	+	−	+	−	+	+	−	+	+	+	+	+	+	+	+	+	+	+
Phenylethylamine	−	+	+	−	−	+	−	+	−	+	+	+	+	+	−	+	+	+
Putrescine	−	−	−	−	−	−	−	−	−	+	+	+	+	+	+	+	+	+
**R**	23	20	24	20	19	24	24	23	24	28	29	28	25	30	26	26	28	28
**AWCD**	0.7	0.3	0.7	0.5	0.4	0.6	0.7	0.6	0.7	1.0	1.1	1.0	0.8	1.1	0.9	1.0	1.0	1.0
**H**′	2.97	2.92	3.05	2.86	2.60	2.96	2.98	2.97	3.03	3.18	3.28	3.17	3.07	3.33	3.11	3.15	3.18	3.19
**E**	0.95	0.97	0.96	0.95	0.88	0.93	0.94	0.95	0.95	0.96	0.97	0.95	0.95	0.98	0.95	0.97	0.96	0.96

**Figure 1:**
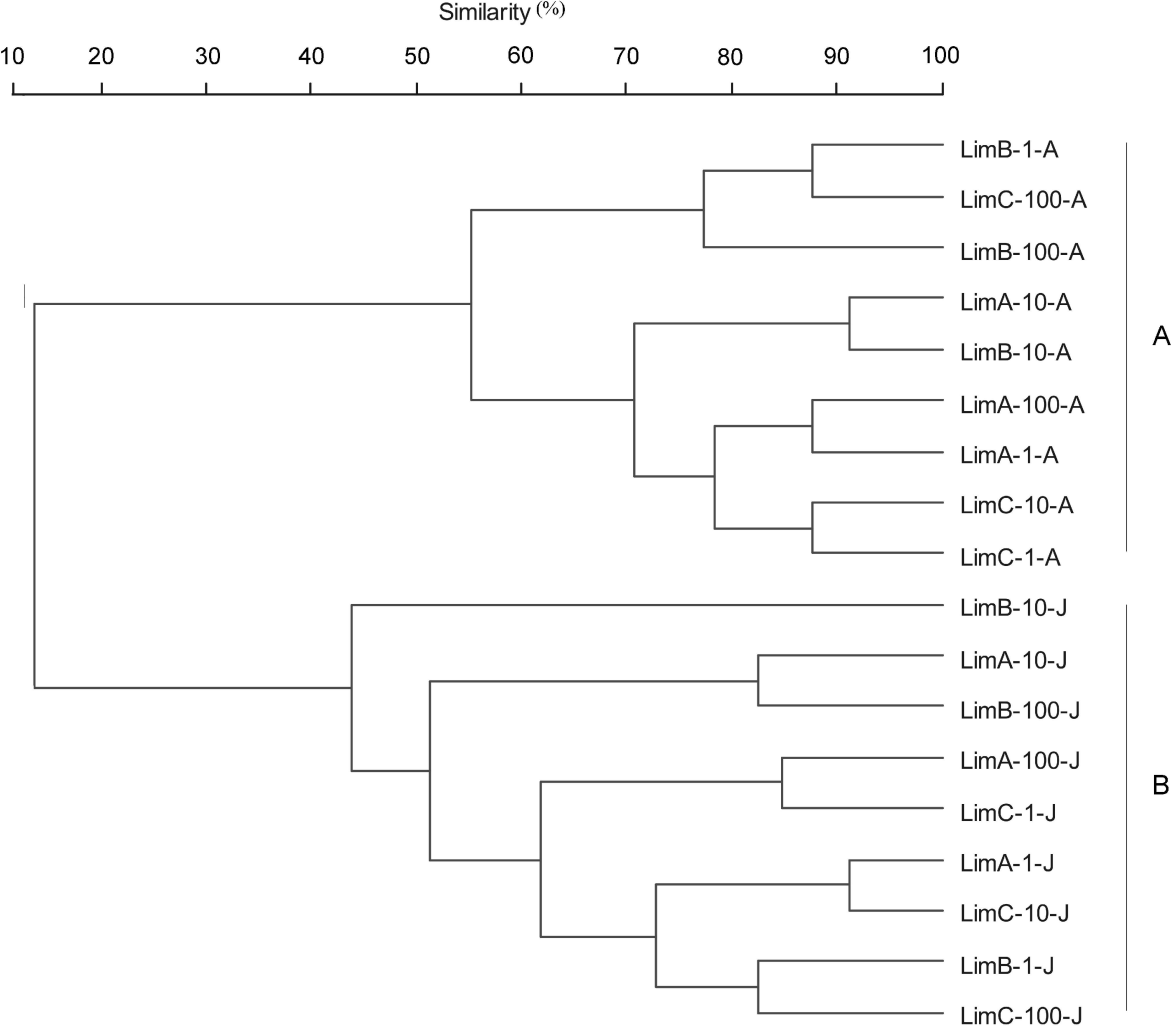
UPGMA cluster analysis of microbial communities based on metabolic diversity. UPGMA (unweighted pair group method using arithmetic averages) cluster analysis of microbial communities based on metabolic diversity obtained through BIOLOG Ecoplates after incubation at 28 °C for 72 h.

The highest functional richness among the 18 microbial communities was detected in the vertical gradient (LimB-10) in August, which utilized 30 C sources. In contrast, in June the LimB-10 sample was the least responsive in the Ecoplate substrate utilization assay and negative for 12 substrates. 2-hydroxy benzoic acid was the single C source that was not utilized by any microbial community obtained from the two sampled periods. Moreover, D-xylose, *γ*-hydroxybutyric acid, and putrescine were not utilized by any microbial community sampled in June. The other C sources were used differentially among the 18 microbial communities (Table 2). Cluster analysis of the Ecoplate data grouped the microbial communities into two principal clusters and revealed that the highest similarity among the microbial communities occurred in August (Fig. 1). The data show a clear separation of the sampled periods, but no pattern in the groupings with respect to horizontal and vertical gradients was observed.

The Ecoplate substrates with greatest number of positive results were complex C sources followed by amino acids, amines, carbohydrates, phosphate–carbon, and carboxylic acids from LimB-10-J, LimB-100-A, LimC-100-A, LimA-100-J, LimB-10-A, and LimC-100-J, respectively (Table 2).

**Figure 2:**
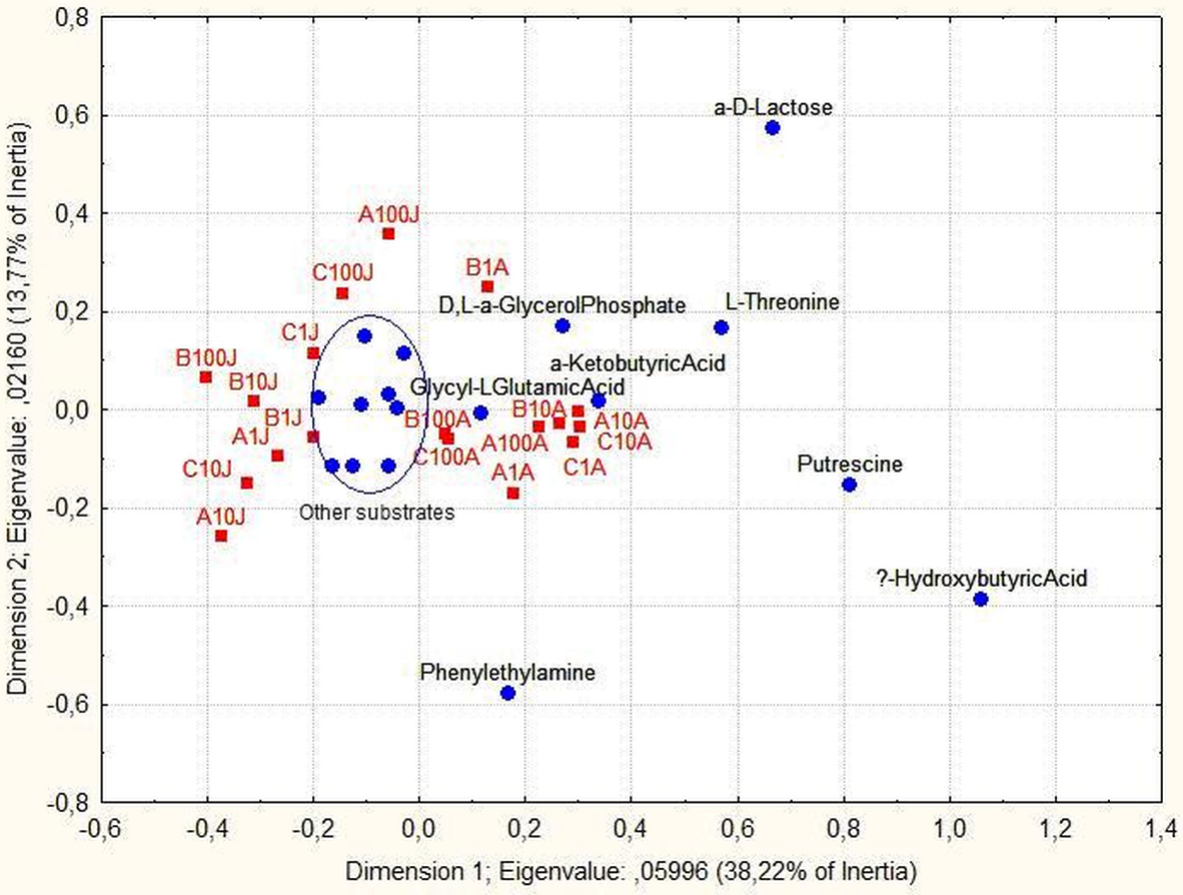
Correspondence analysis. Projection of substrates and samples on the 1–2 factorial plane. In blue the substrates and in red the sampling points.

Multivariate analysis, CA, demonstrated responses of the microbial communities induced by the Ecoplate substrates (Fig. 2). The axis I differentiated (38.22%) the samples according to the period of the year. The microorganisms contained in the samples from August (positive coordinates of axis I) were able to degrade hydroxybutiric acid, putrescine, L-threonine, D, L-*α*-glycerol phosphate, *α*-ketobutyric acids, glycyl-L-glutamic acid and phenylethylamine. This group of substrates was typically used in August, but not in June. For the second dimension of the CA only 13.8% of the variation is explained, with a higher diversity of substrate usage according to the depth of sampling. The substrate *α*-D-lactose was used by the microbial community from the point corresponding to 100% of light penetration in June, whereas phenylethylamine was used by the microbial communities of the surface waters (1% and 10 % of light penetration).

### Recovery of isolates and their taxonomic assignment

Results from direct cultivation of bacteria from horizontal and euphotic gradients of the limnetic zone on PTYG agar showed low isolate recovery, ranging from 1.2 × 10^2^ CFU ml^−1^ (June) to 3.8 × 10^2^ CFU ml^−1^ (August). After purification of the colonies, a total of 673 bacterial isolates were identified by 16S rRNA gene fragment sequencing (LimA 122 and 128, LimB 109 and 113, and LimC 82 and 119 in June and August, respectively).

**Figure 3:**
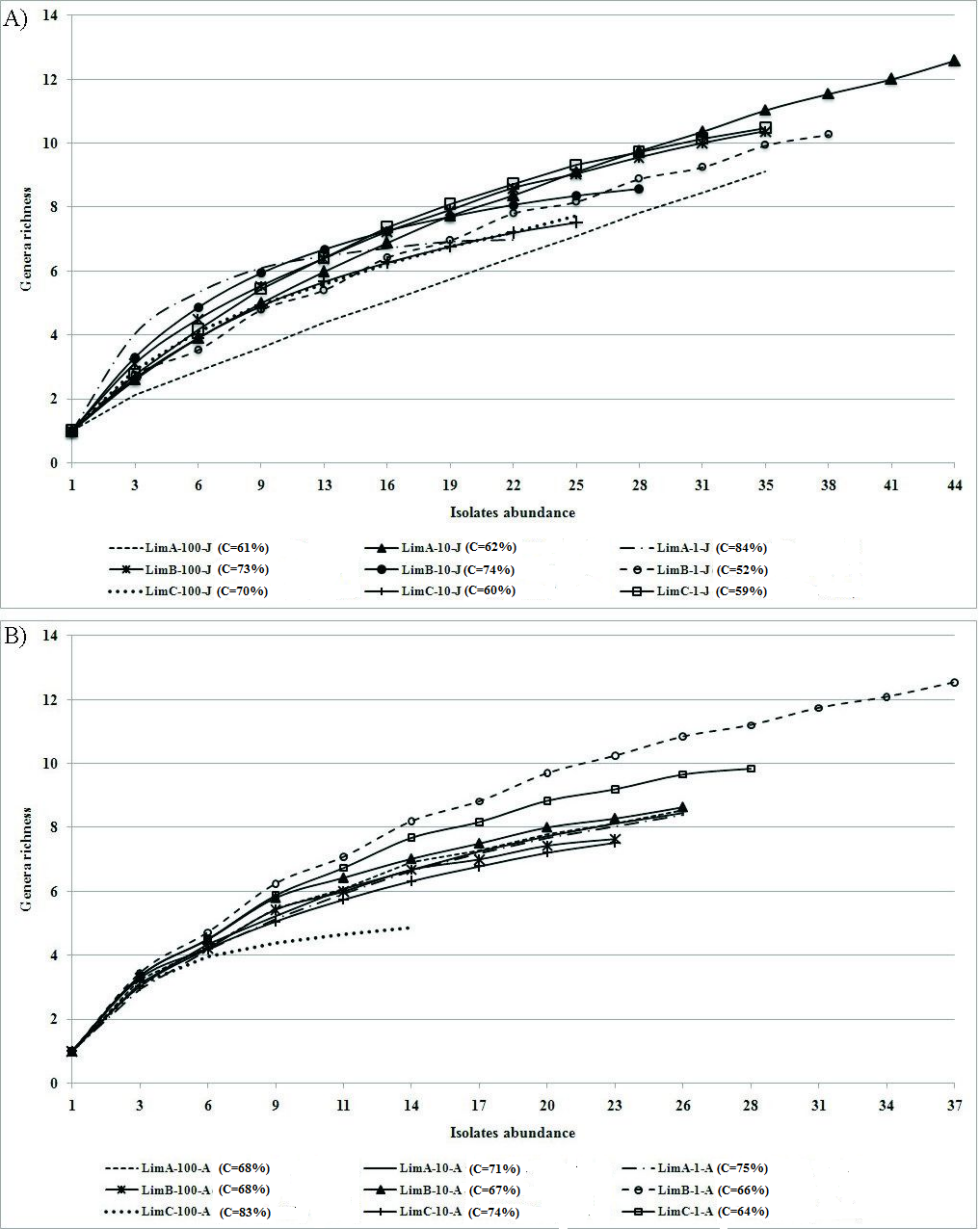
Rarefaction curves for each bacterial community based on the 16S rRNA gene sequences. C, coverage. (A) bacterial communities from June; (B) bacterial communities from August.

Rarefaction curves were used to determine whether the sampling was enough to evaluate genera diversity with some level of confidence. Rarefaction curves were obtained by plotting the genera richness observed against the number of isolates analyzed for each of the 18 communities (Figs. 3A and 3B). A decrease in the rate of genera detection was observed in the bacterial communities from August, except for LimC-1-A (coverage = 64%; Fig. 3B). In June, a decrease was observed in five of the nine bacterial communities, indicating that the majority of the diversity was detected. This result was further supported by calculating the coverage of the communities (≥62%; Fig. 3A).

The ARDRA data from all examined isolates obtained with two restriction endonucleases (AflIII and AluI) were combined into a single restriction pattern for each isolate. The number of restriction fragments for AflIII ranged from one to eight and for AluI from one to nine (data not shown). The analysis of combined restriction patterns revealed 360 distinct patterns. Operational Taxonomic Units (OTUs) were defined as unique ARDRA patterns. The ARDRA patterns were primarily composed of single-isolate OTUs (252 of 360). Three hundred and thirteen of the OTUs were from isolates obtained in a single sampling point, while the remaining 47 OTUs were from isolates obtained at multiple points. Moreover, the temporal ARDRA OTU distribution showed that only 13 of them were detected in both collection months (Table 3).

**Table 3:** Spatial distribution ARDRA OTUs and statistical diversity of bacterial isolates communities.

Euphotic and Horizontal Gradients	Number of isolates	Number of unique ARDRA OTUs	ARDRA OTUs repeated between:	
			Euphotic and Horizontal Gradients	Month of collection
LimA-100-J	36	18	1	1
LimA-10-J	37	22	6	2
LimA-1-J	49	35	1	0
LimB-100-J	44	26	0	2
LimB-10-J	38	20	0	3
LimB-1-J	27	19	1	0
LimC-100-J	29	13	1	2
LimC-10-J	22	13	2	2
LimC-1-J	31	17	0	1
LimA-100-A	36	12	7	0
LimA-10-A	49	16	1	0
LimA-1-A	43	22	2	0
LimB-100-A	40	13	6	0
LimB-10-A	35	7	1	0
LimB-1-A	38	22	1	0
LimC-100-A	39	16	2	0
LimC-10-A	47	11	2	0
LimC-1-A	33	11	0	0
Total	673	313	34	13

**Notes.**

OTUs Operational Taxonomic Units ARDRA Amplified Ribosomal DNA Restriction Analysis Lim Limnetic zone

The numbers 1, 10 and 100 correspond to light penetration (1%, 10% and 100%) as determined by Secchi disk.

The letters A, B and C correspond to each point of the limnetic zone that the isolates were recovered.

The letters J and A correspond to month of collection (J for June and A for August).

**Figure 4:**
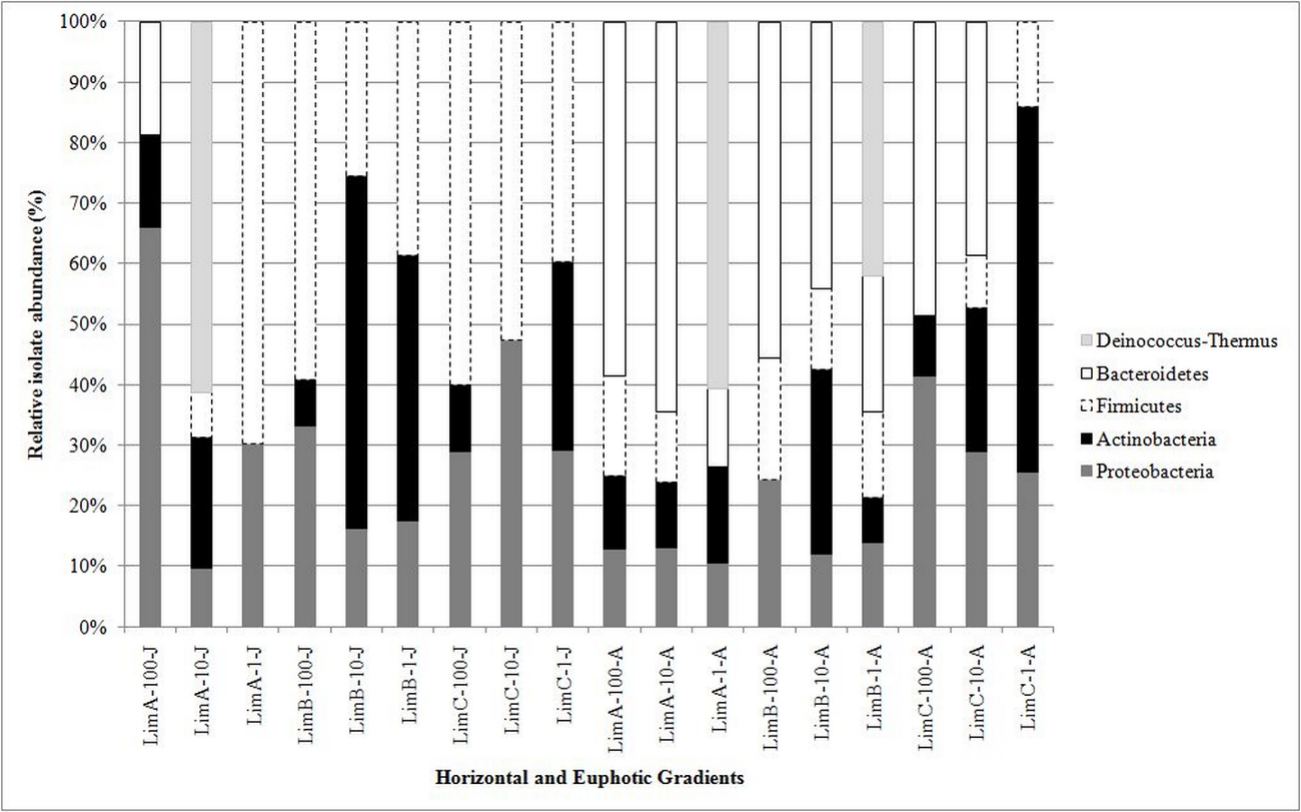
Relative abundance of phyla in Carioca Lake based on sequence analysis of the 16S rRNA gene.

**Figure 5:**
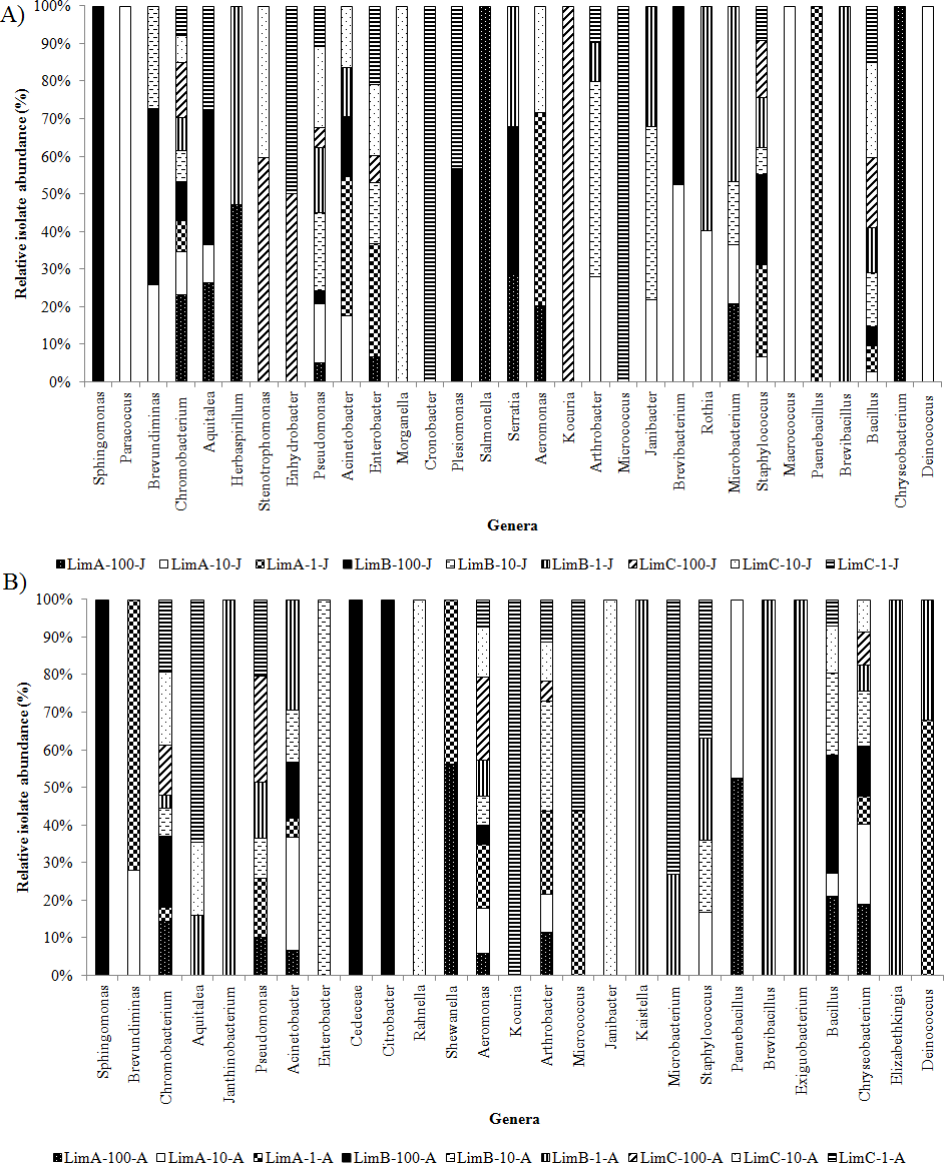
Relative abundance of genera in Carioca Lake based on sequence analyses of the 16S rRNA gene. (A) bacterial communities from June; (B) bacterial communities from August.

All sequences, corresponding to variable V2–V4 regions of 16S rRNA gene were categorized into five phyla: *Proteobacteria* (61.8%, includes *Alpha*-, *Beta*-, and *Gamma-proteobacteria*), *Firmicutes* (18.6%), *Actinobacteria* (10.2%), *Bacteroidetes* (8.5%), and *Deinococcus*-*Thermus* (0.9%). The distribution and abundance of the five bacterial phyla (Fig. 4) differed in all bacterial communities from June and August (*p* < 0.01). *Proteobacteria* was consistently found in all communities and in both collection months. In contrast, *Bacteroidetes* was only detected in LimA-100-J and in eight out of the nine communities from August. Members of *Firmicutes* and *Actinobacteria* were also cosmopolitan. *Deinococcus*-*Thermus* was distributed only at three points at ≤10% of light penetration. Consequently, the distribution of the bacterial genera also differed considerably among the 18 bacterial communities (Fig. 5).

Sequences were recovered from 38 genera from all five phyla (Figs. S2–S5 and Table S1). For *Proteobacteria*, sequences represented 21 genera from *Gamma-proteobacteria* (53%), *Beta-proteobacteria* (43.2%), and *Alpha-proteobacteria* (3.8%) (Table S1). Among the 38 genera, *Chromobacterium* was the most common genus, missing only from LimA-10-A. Other common genera were: *Pseudomonas*, *Bacillus*, *Acinetobacter*, *Arthrobacter* and *Staphylococcus*. Some genera were detected in only one of the collection months. In June we detected the genera *Paracoccus*, *Herbaspirillum*, *Stenotrophomonas*, *Enhydrobacter*, *Morganella*, *Cronobacter*, *Plesiomonas*, *Serratia*, *Janibacter*, *Brevibacterium*, *Rothia* and *Macrococcus* (Fig. 5A). In August, we detected *Janthinobacterium*, *Cedecea*, *Citrobacter*, *Rahnella*, *Shewanella*, *Kaistella*, *Exiguobacterium* and *Elizabethkingi*a (Fig. 5B). Likewise, some genera from each sampling period were restricted to a specific euphotic gradient point, predominantly at 1% and 10% of light penetration. Interestingly, *Sphingomonas* (LimB-100-J) and *Brevibacillus* (LimB-1-A) were restricted to the same horizontal and vertical gradient points in both months (Fig. 5).

*Bacteroidetes* and *Deinococcus*-*Thermus* were less represented, with few genera in each phylum. The isolates associated with *Bacteroidetes* were represented by the genera *Chryseobacterium*, *Kaistella* and *Elizabethkingia*, and *Deinococcus* represented the *Deinococcus*-*Thermus* phylum.

**Figure 6:**
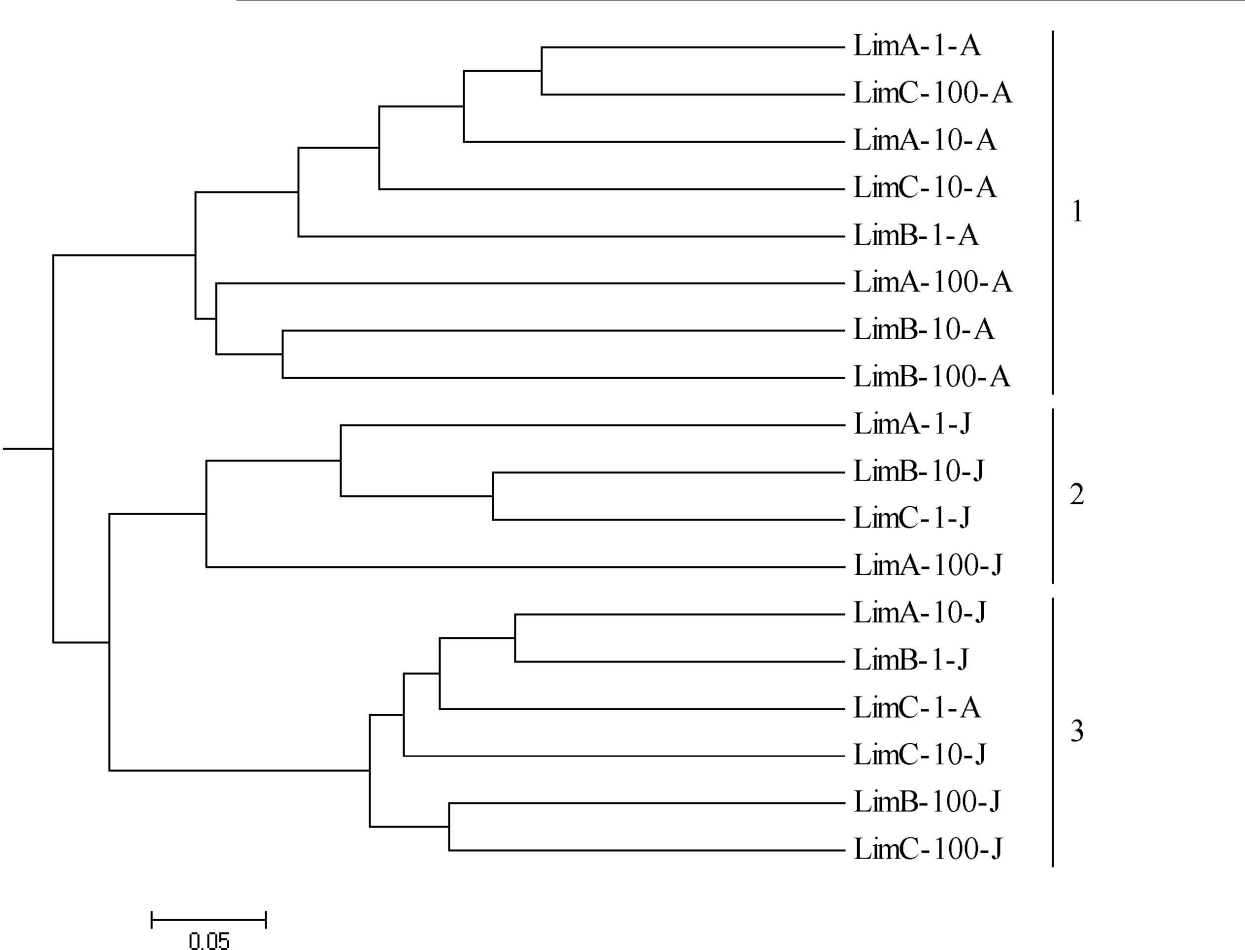
UPGMA (unweighted pair group method using arithmetic averages) dendrogram generated by UniFrac of isolates from 18 bacterial communities from Carioca Lake.

To compare the composition of bacterial communities and specific features of the ecosystems, we used the UniFrac metric analysis. The data revealed three main clusters of related communities. Cluster 1 matches the August collection period, except for Lim-C-1-A, which is located in cluster 3. Clusters 2 and 3 combined the isolates from June (Fig. 6). The communities were grouped with low similarities, showing the difference of genera composition. The robustness of the inferred UniFrac tree topology to the presence of specific isolates represented was confirmed by jackknife analysis (*p* < 0.01).

**Figure 7:**
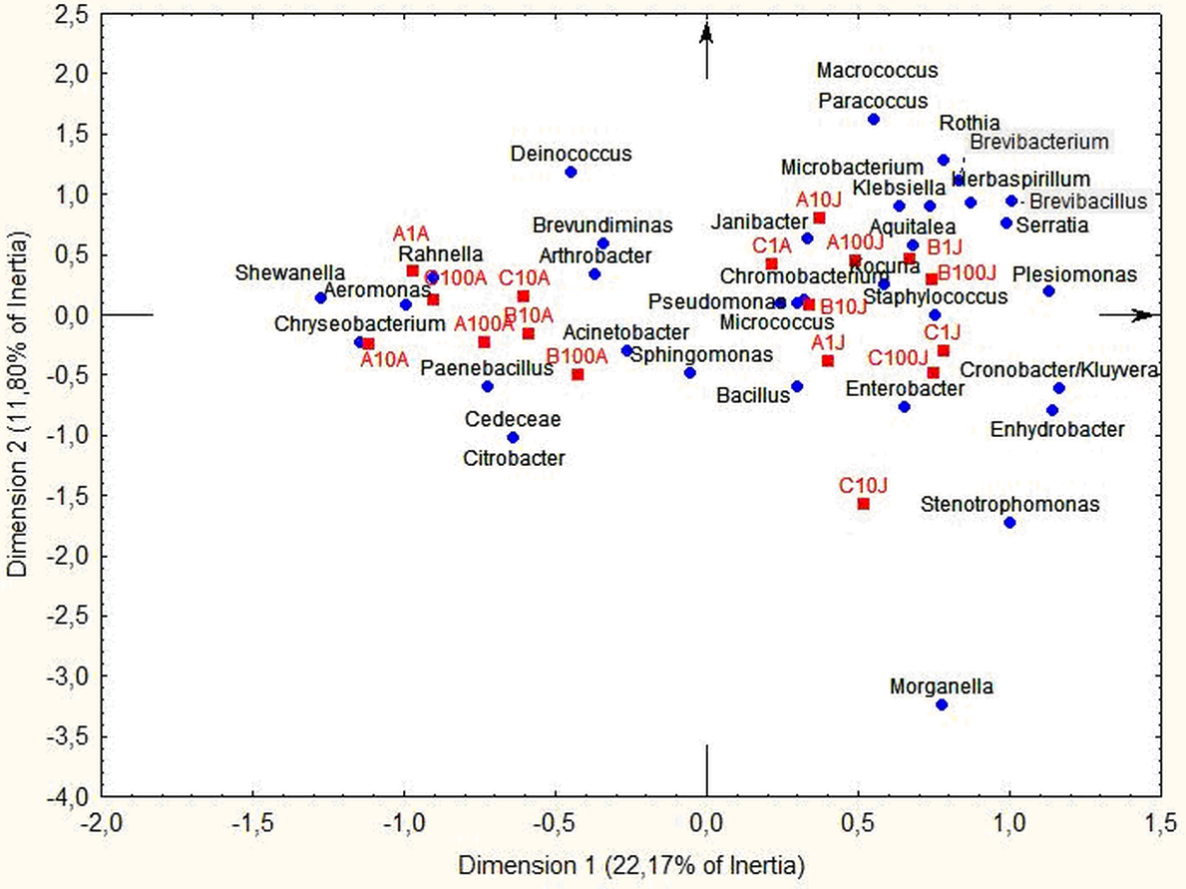
Correspondence analysis. Projection of bacteria and samples on the 1–2 factorial plane. The samples are in red, the genera are in blue. (LimB-1-A sample was excluded—see text).

We also performed CA with the genera frequency and the Ecoplate substrates usage frequency, removing the sample LimB-1-A, as it distorted the grouping (Fig. 7). The first dimension separated the sampling periods (22.17%), August placed in the negative coordinate and June in the positive coordinate. The sample LimC-1-A was an exception with the presence of *Chromobacterium* in both months. *Shewanella*, *Chryseobacterium*, *Paenebacillus*, *Cedeceae*, *Citrobacter* and *Aeromonas* occurred in August, while *Morganella*, *Stenotrophomonas*, *Enhydrobacter*, *Cronobacter* and *Enterobacter* occurred in June. In the stations A and B we found *Serratia*, *Brevibacillus*, *Herbaspirillum*, *Brevibacterium*, *Rothia*, *Macrococcus* and *Paracoccus*. The Lim-C sample had highest bacterial richness, but no clear difference between the depths. Moreover, it was observed that there was a probable association between taxonomic groups and some substrates, e.g., *Acinetobacter*, *Aeromonas* and *Chryseobacterium* had preference for *α*-D-Lactose, Υ-hydroxybutyric acid, *α*-ketobutyric acid, L-threonine, putrescine and phenylethylamine (Fig. 7).

## Discussion

When compared to the temperate lentic ecosystems, tropical freshwater lake bacteria have been scarcely studied either by culture-independent or dependent methods. Therefore, in this study temporal and spatial molecular and physiological culture-dependent investigations were performed to shed light into of the phylogeny of tropical freshwater heterotrophic bacteria.

BIOLOG Ecoplate has been recognized as a useful tool to study functional diversity and to compare bacterial communities (Smalla et al., 1998). CLPP reflect the potential of the bacterial community to respond to different C sources. According to Chazarenc, Brisson & Merlin (2010), complex C sources allow the best growth of bacterial communities because microorganisms can conserve more energy when catabolizing these compounds. In the present study, CLPPs revealed differences in the functional diversity of the microbial communities in June and in August. The 18 bacterial communities studied here showed high AWCD and R values. These communities greatly metabolized complex C sources (Tween 80, Tween 40, *α*-cyclodextrin, and glycogen). The dendrogram and correspondence analysis plot from the metabolic profile revealed clearly distinct bacterial communities in the sampling periods, suggesting changes in the bacterial community. Although ARDRA is used as a shortcut to minimize sequencing of clone libraries, it does not allow for the identification of specific phylogenetic groups within a community profile. In this study, although ARDRA were distinct for most isolates, showing 313 unique OTUs (Table 3), several ARDRA OTUs were found representing the same genus. Analyzing 16S rRNA gene clone libraries by ARDRA, Dang & Lovell (2000) found distinct OTUs harboring closely related sequences. Therefore, this approach reveals the number of different 16S rRNA genes retrieved from a sampling site, but not the number of different varieties of organisms in the sample. Moreover, these genetic variants could represent ecotypes, reflecting physiological adaptations to their habitats (Acinas, Antón & Rodríguez-Valera, 1999; Oda et al., 2003). Differences in the composition of bacterial communities, especially in relation to the composition of ecotypes and the number of isolates, were also found in other environments (Oda et al., 2003).

Despite the fact that cultivation techniques are restrictive in recovering bacterial isolates from environmental samples (Torsvik & Øvreås, 2002) the use of PTYG medium was effective in recovering a wide phylogenetic range of freshwater bacteria, including a rare genus, *Cedecea*. These data were supported by the coverage values (52%–84%), showing that the diversity found has an appropriate level of confidence. Moreover, our findings are in agreement with Berg et al. (2009) and Vaz-Moreira et al. (2011), who found the same phyla in the isolation of heterotrophic bacteria from a variety of water sources, being that most genera found were coincidently detected in our study. It is noticeable that Berg et al. (2009) using five different growth media, recovered a total of 53 genera. From the high productivity medium, 28 different genera were isolated, whereas the minimum medium gave six different genera. In contrast, our study recovered 38 genera using only a culture medium. It should be noted that the use of media with lower nutrient concentrations increase the isolation efficiency as well as the diversity of bacteria from an environmental sample (Puspita et al., 2012). Although the choice of the high nutrient medium (PTYG) is not the best option for the isolation of environmental bacteria, it was suitable for an initial assessment of the bacterial diversity in our study.

The 38 bacterial genera to which the isolates were assigned, based on partial sequence analysis of 16S rRNA genes, belonged to five bacterial phyla: *Proteobacteria* (especially *Gamma-proteobacteria*), *Firmicutes*, *Actinobacteria*, *Deinococcus-Thermus* and *Bacteroidetes*. Among these phyla, *Proteobacteria* (especially *Beta-proteobacteria*), *Actinobacteria* and *Bacteriodetes* are considered typical freshwater bacteria (Zwart et al., 2002). In our study, a higher abundance of the 16S rRNA gene sequences of the *Firmicutes* and *Deinococcus-Thermus* phyla were recovered, both of which have been shown to occur only sparsely in freshwater environments (Logue & Bürgmann Robinson, 2008). However, the in-depth phylogenetic analysis reported herein revealed that not all isolates recovered are typical freshwater genera.

The predominance of members of the *Gamma-proteobacteria* class is interesting since it is normally found in lesser abundance in freshwater lakes (Newton et al., 2011). Representatives of this class are copiotrophic, organisms found in nutrient-rich environments (Zavarzin, Stackebrandt & Murray, 1991), and therefore often recovered from lakes that are highly productive or polluted (Ghai et al., 2011), being also often enriched in culture-dependent studies in high nutrient medium. However, it should be noted that Carioca Lake has not experienced anthropogenic activities and is hydrologically isolated from the original main drainage (River Doce), which is at present approximately 40 m deeper than the lakes formed in its paleo-canal.

In both sampling months, the environmental parameters obtained in the water column presented little variation that could explain the distinct taxonomically and functionally bacterial communities that were found. Other studies also observed changes in the freshwater lake bacterial community composition in different periods. Nevertheless, some genera, such as *Pseudomonas*, *Acinetobacter* and *Enterobacter*, were consistently recovered in freshwater lakes (Pontes et al., 2009; Czekalski et al., 2012).

The present study detected 38 genera, which are widely distributed in the environment, having been isolated from soil, water, and clinical material (Zwart et al., 2002; Berg et al., 2009; Humbert et al., 2009; Toh et al., 2010; Lee et al., 2011; Yang & Li, 2011; Soltan Dallal, Sharifi Yazdi & Avadisians, 2011; Costa et al., 2012). Among the genera, *Aeromonas*, *Chromobacterium*, *Brevundimonas*, *Sphingomonas*, *Staphylococcus*, *Bacillus*, *Arthrobacter* and *Microbacterium* predominated in one or both sampling months, with wide variation of their frequencies, except for *Brevundimonas*. In a previous culture-dependent study, Berg et al. (2009) found genera similar to those described in our study when investigating heterotrophic bacteria associated with cyanobacterial growth. The report revealed that many of these genera were able to inhibit or enhance cyanobacterial growth and suggested that they can be used in assessing and controlling the harmful effects of cyanobacteria.

This is the first study with a large set of heterotrophic bacteria from a tropical freshwater lake that had not experienced anthropogenic activities. In conclusion, our results provide evidence of complex heterothrophic bacterial communities in a tropical freshwater lake, which seem well adapted to different nutrients provided by the lake and litter therein. The conditions employed evidence of the predominance of large taxonomic groups of bacteria, *Gamma-proteobacteria* and *Firmicutes*, which are otherwise found in low abundance in freshwater lakes. Taken together, our data expand the current knowledge about the diversity of tropical freshwater lake bacteria, which are not fully explored and deserve further attention.

## Supplemental Information

10.7717/peerj.478/supp-1 Figure S1 Rio Doce State Park Rio Doce State Park and part of the lake system evidencing the sampling site location, Carioca Lake.

10.7717/peerj.478/supp-2 Figure S2 Phylogenetic ARB affiliation Neighbor-joining phylogenetic ARB affiliation of *Alpha*- and *Beta*- *proteobacteria* based on 16S rRNA gene sequences. The numbers in parentheses indicate the number of isolates in each haplotype. Bootstrapping was performed with 500 replications. *Thermodesulfatator indicus* DSM 15286 (accession number CP002683) was used as outgroup. Scale bar: 0.1 substitutions per site.

10.7717/peerj.478/supp-3 Figure S3 Phylogenetic ARB affiliation Neighbor-joining phylogenetic ARB affiliation of *Gamma*-*proteobacteria* based on 16S rRNA gene sequences. The numbers in parentheses indicate the number of isolates in each haplotype. Bootstrapping was performed with 500 replications. *Thermodesulfatator indicus* DSM 15286 (accession number CP002683) was used as outgroup. Scale bar: 0.1 substitutions per site.

10.7717/peerj.478/supp-4 Figure S4 Phylogenetic ARB affiliation Neighbor-joining phylogenetic ARB affiliation of *Firmicutes* based on 16S rRNA gene sequences. The numbers in parentheses indicate the number of isolates in each haplotype. Bootstrapping was performed with 500 replications. *Thermodesulfatator indicus* DSM 15286 (accession number CP002683) was used as outgroup. Scale bar: 0.1 substitutions per site.

10.7717/peerj.478/supp-5 Figure S5 Phylogenetic ARB affiliation Neighbor-joining phylogenetic ARB affiliation of *Actinobacteria, Bacteroidetes and Deinococcus-Thermus* based on 16S rRNA gene sequences. The numbers in parentheses indicate the number of isolates in each haplotype. Bootstrapping was performed with 500 replications. *Thermodesulfatator indicus* DSM 15286 (accession number CP002683) was used as outgroup. Scale bar: 0.1 substitutions per site.

10.7717/peerj.478/supp-6 Table S1 Taxonomic affiliation Taxonomic affiliation and distribution of bacterial isolates from Carioca Lake.

## Additional Information and Declarations

### Field Study Permissions

The following information was supplied relating to field study approvals (i.e., approving body and any reference numbers): All necessary permits were obtained from the Instituto Estadual de Florestas (no. 018/2008) for the field studied.

### DNA Deposition

The following information was supplied regarding the deposition of DNA sequences: The nucleotide sequences generated were deposited in the GenBank database with accession numbers JF900768–JF901324.
