# Supplementary figures and images for: A survey on cultivable heterotrophic bacteria inhabiting a thermally unstratified water column in an Atlantic Rainforest lake
Source: PeerJ. 2014 Aug 26;2:e478. doi: 10.7717/peerj.478 (PMC4157234; doi:10.7717/peerj.478)

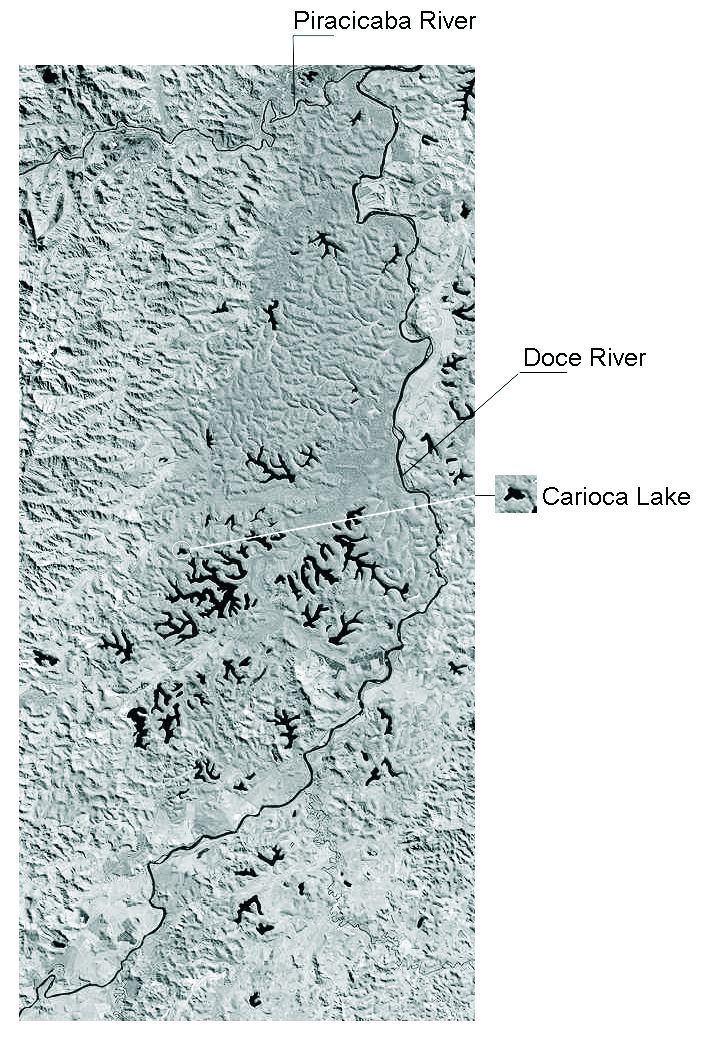

Supplement: Figure S1 — Rio Doce State Park and part of the lake system evidencing the sampling site location, Carioca Lake. [file peerj-02-478-s001.jpg]

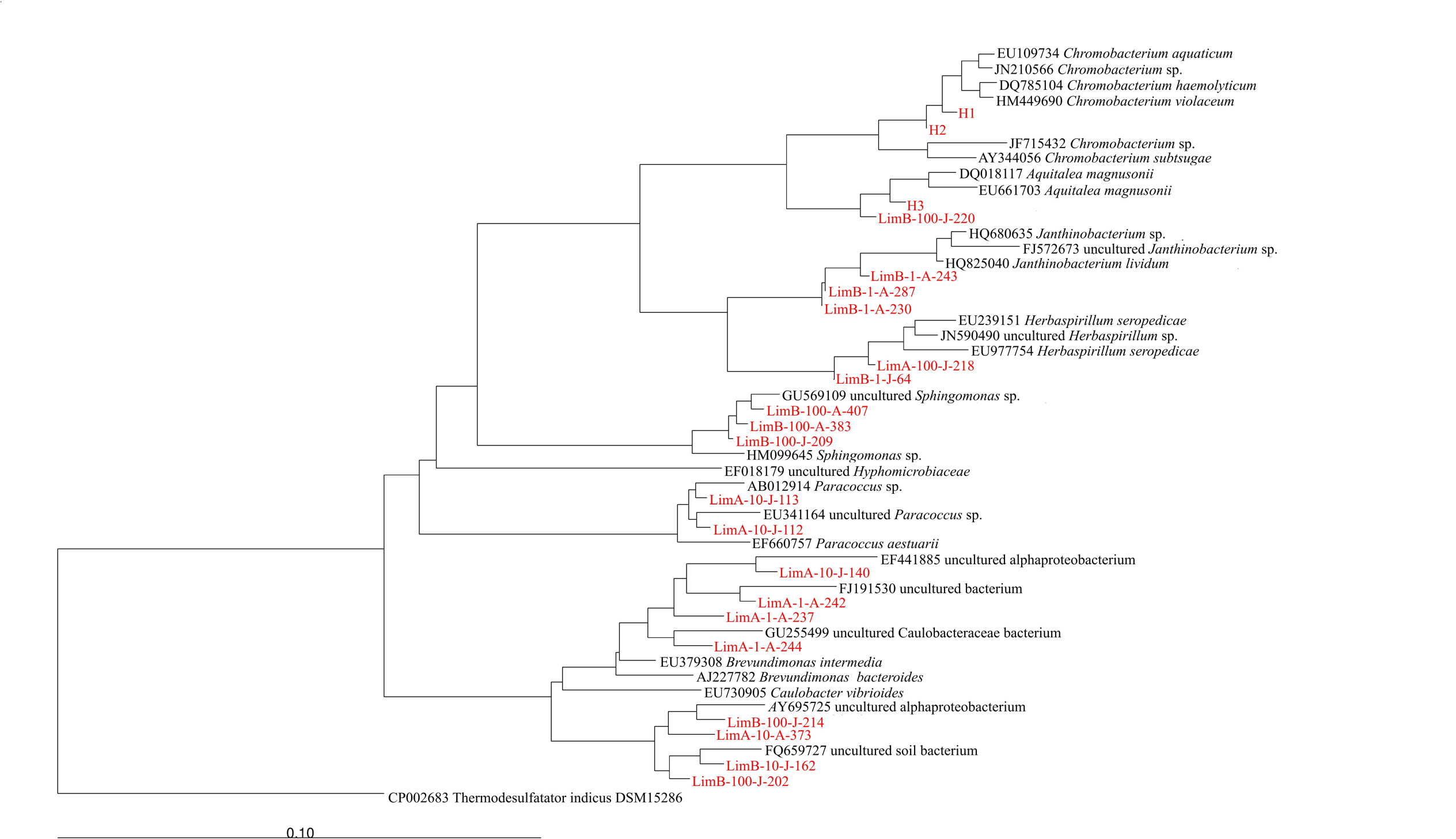

Supplement: Figure S2 — Neighbor-joining phylogenetic ARB affiliation of Alpha- and Beta- proteobacteria based on 16S rRNA gene sequences. The numbers in parentheses indicate the number of isolates in each haplotype. Bootstrapping was performed with 500 replications. Thermodesulfatator indicus DSM 15286 (accession number CP002683) was used as outgroup. Scale bar: 0.1 substitutions per site. [file peerj-02-478-s002.jpg]

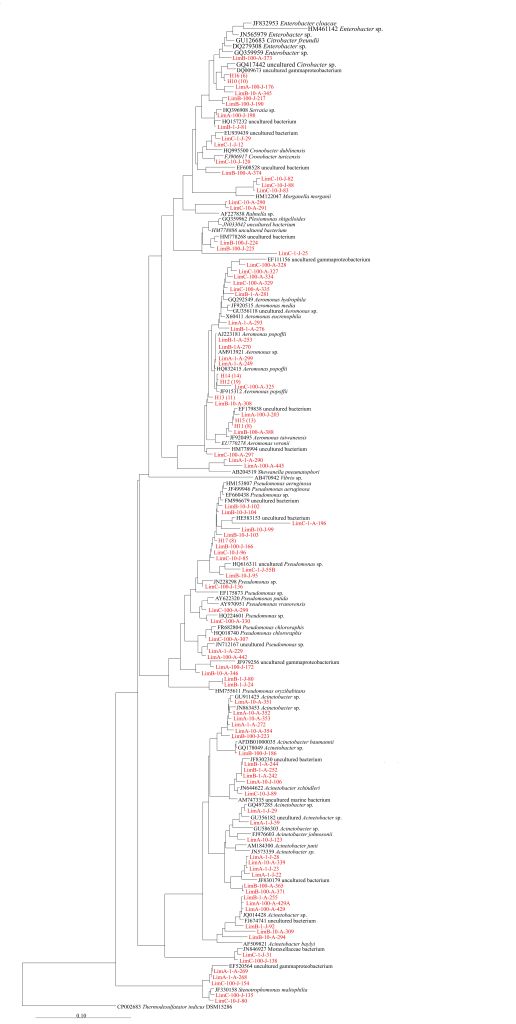

Supplement: Figure S3 — Neighbor-joining phylogenetic ARB affiliation of Gamma-proteobacteria based on 16S rRNA gene sequences. The numbers in parentheses indicate the number of isolates in each haplotype. Bootstrapping was performed with 500 replications. Thermodesulfatator indicus DSM 15286 (accession number CP002683) was used as outgroup. Scale bar: 0.1 substitutions per site. [file peerj-02-478-s003.jpg]

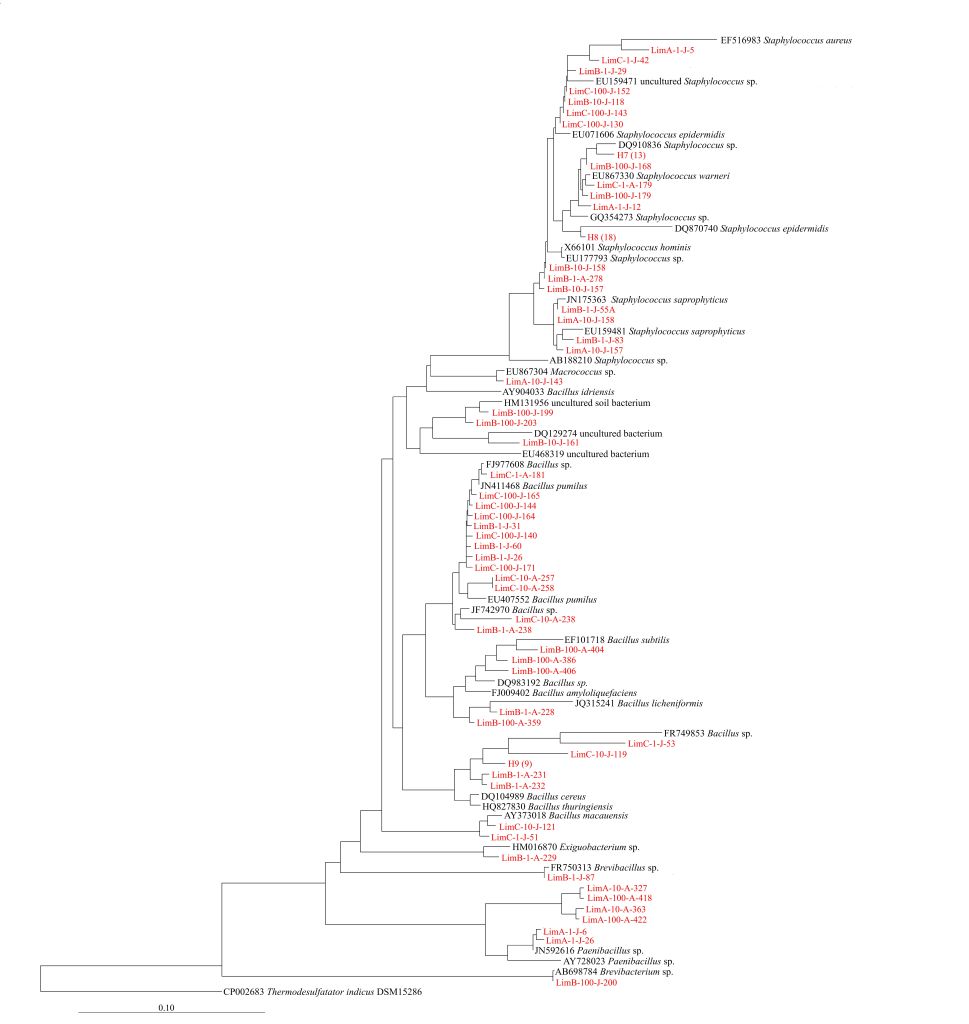

Supplement: Figure S4 — Neighbor-joining phylogenetic ARB affiliation of Firmicutes based on 16S rRNA gene sequences. The numbers in parentheses indicate the number of isolates in each haplotype. Bootstrapping was performed with 500 replications. Thermodesulfatator indicus DSM 15286 (accession number CP002683) was used as outgroup. Scale bar: 0.1 substitutions per site. [file peerj-02-478-s004.jpg]

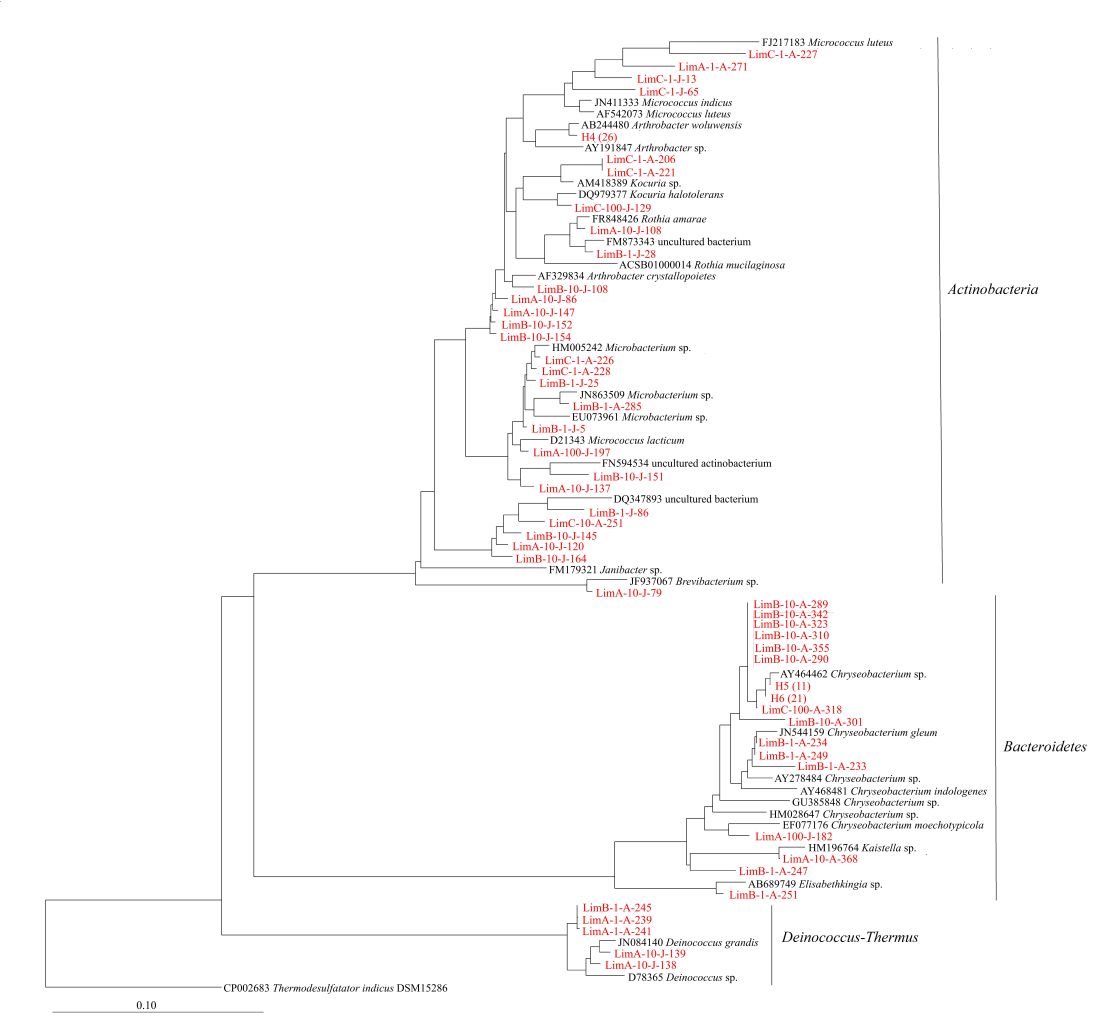

Supplement: Figure S5 — Neighbor-joining phylogenetic ARB affiliation of Actinobacteria, Bacteroidetes and Deinococcus-Thermus based on 16S rRNA gene sequences. The numbers in parentheses indicate the number of isolates in each haplotype. Bootstrapping was performed with 500 replications. Thermodesulfatator indicus DSM 15286 (accession number CP002683) was used as outgroup. Scale bar: 0.1 substitutions per site. [file peerj-02-478-s005.jpg]
